# A Neuronal Network Model for Simulating the Effects of Repetitive Transcranial Magnetic Stimulation on Local Field Potential Power Spectra

**DOI:** 10.1371/journal.pone.0049097

**Published:** 2012-11-07

**Authors:** Alina Bey, Stefan Leue, Christian Wienbruch

**Affiliations:** 1 Department of Computer and Information Science, University of Konstanz, Konstanz, Germany; 2 Department of Psychology, University of Konstanz, Konstanz, Germany; University of Regensburg, Germany

## Abstract

Repetitive transcranial magnetic stimulation (rTMS) holds promise as a non-invasive therapy for the treatment of neurological disorders such as depression, schizophrenia, tinnitus, and epilepsy. Complex interdependencies between stimulus duration, frequency and intensity obscure the exact effects of rTMS stimulation on neural activity in the cortex, making evaluation of and comparison between rTMS studies difficult. To explain the influence of rTMS on neural activity (e.g. in the motor cortex), we use a neuronal network model. The results demonstrate that the model adequately explains experimentally observed short term effects of rTMS on the band power in common frequency bands used in electroencephalography (EEG). We show that the equivalent local field potential (eLFP) band power depends on stimulation intensity rather than on stimulation frequency. Additionally, our model resolves contradictions in experiments.

## Introduction and Related Work

### Influence of rTMS on motor cortex areas

Concerning the repetitive application of transcranial magnetic stimulation (rTMS), it has been suggested that low stimulation frequencies (≤1 Hz) have lasting inhibitory effects at least on motor cortex areas [1], while higher rTMS frequencies of 5 Hz and 10 Hz induce an overall increase in motor excitability [2], [3]. But combined EEG-TMS studies indicate that a simple dichotomous influence of high and low frequencies is too reductive [4]. Several studies report a decrease in the amplitude of the motor evoked potentials (MEP) after low-frequency rTMS stimulation [3], [5]. At high stimulation frequencies some studies [6] report an increase in MEP amplitude while others observe the opposite [4]. These discrepancies are explained by the high variability among individual subjects within each study and the differing stimulation intensities used in the studies [4], [6], [7].

Furthermore, broad consensus has not yet been established regarding the dependency of the rTMS stimulation frequency on EEG band power. While some studies report no significant changes in the alpha band after 10 Hz rTMS stimulation [8], [9], others find enhanced alpha activity after the application of 10 Hz rTMS [10] or a decrease in alpha activity after the application of 5 Hz rTMS [11]. At least, the effect of rTMS on the activity in the gamma band seems undisputed: a significant increase in gamma band activity has been observed in healthy individuals after 20 Hz rTMS [12], [13], [14].

The influence of rTMS on the power in the delta band is also subject to experimental research. Activity increases in the delta band after the application of 10 Hz rTMS are stated [8].

### Models of neural activity

In order to understand the influence of stimulus frequency and stimulus intensities on EEG measurements, we provide a neuronal network model which allows for the simulation of rTMS and the observation of its effects after the end of the stimulation period.

Such networks have been intensively examined in the past few decades, resulting in different insights into network behaviour and a broad range of different models. While physical models focus mainly on the oscillatory behaviour of the model components (e.g. the Kuramoto oscillator) [15], approaches based on graph theory allow elaboration on the stochastic properties [16] of complex networks [17] and are useful to explore the global structure of the network.

Some of the existing models already integrate the effect of TMS on the network behaviour, but they differ in their degree of abstraction. Detailed models represent the behaviour of the neuronal entities and the TMS induced axial and transmembrane currents by sets of differential equations [18], their output being focussed on the influence of the TMS induced activity on the neuronal spike trains [18]. A more abstract model is described in [19]: four different types of neuronal units constitute a simplified cortical column. A TMS stimulus is simulated by increasing current in the selected regions of the model. The number of active regions determines the functional connectivity of the brain regions [19]. Obviously, these models do not allow any conclusions about the effect of rTMS on eLFP band power.

**Figure 1:**
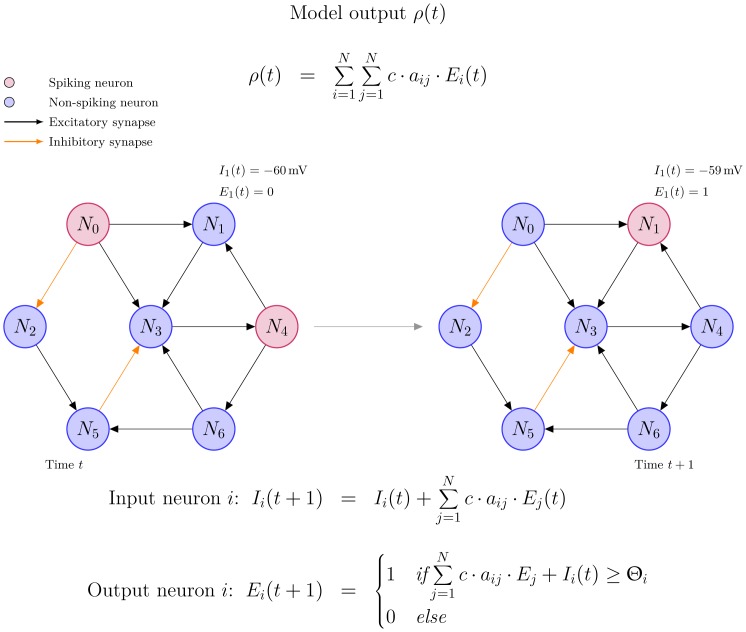
Example cluster of 7 neurons for the time evolution of our model (left panel: time t = 1 and right panel: t = t+1. Spiking neurons are coloured red, non-spiking neurons are coloured blue. Synaptic connections between the neurons are either excitatory (black) or inhibitory (orange). The functions I_i_(t) and E_i_(t) describe the input and output of each neuron. The threshold Θ_i_ triggers the generation of a spike and is set to −60 mV. The constant c is denoting the change in postsynaptic potential triggered by the arrival of an action potential (AP) at the synaptic cleft. The model output ρ(t) consists of the sum of the output E_i_(t) of all neurons.

We define a random graph based asymmetric Hopfield network with synchronous updates [20], [21]. Figure 1 shows a schematic view of the model and its behaviour over time. With our model, we are able to validate observations made during *in vivo* rTMS experiments leading to the assumption that the simulation is realistic. Furthermore, the model helps to understand the influence of the intensity and frequency of the stimulation protocol on EEG power spectra and thus may help in reconciling the discrepancies between the aforementioned studies.

## Results

### Influence of network size

We state that the network size (1000, 2000, 4000, 8000 and 16000 neurons) has no significant influence on the band power. Instead, we identify the average number of synaptic connections maintained by each neuron as dominant parameter. Our numerical analysis of the network behaviour showed that an average node degree k = 120 could explain best the EEG measurements of the motor cortex areas subject to rTMS stimulation. We therefore assume that our model represents the effect of rTMS stimulation on the motor cortex.

### Statistics

The effects are summarized in Table 1. All reported effects are short term effects and refer to the first 1000 ms after the end of the stimulation. We stated a significant main effect for factor TIME (F(1,16) = 574.7, p<5.8e-14), a larger mean power before than after the rTMS stimulation (mean and standard error of mean: PR E 5.54±0.006; POST 5.44±0.008) and a significant main effect for the factor FREQUENCY-BAND F(4.64) = 21626, p<0.0000 (delta band: 6.27±0.01, theta band 6.05±0.01, alpha band 5.71±0.01, beta band 5.44±0.005, gamma band 3.98±0.004), constantly decreasing with increasing band frequency.

**Table 1:** Results of the repeated measures ANOVA: full factorial general linear model.

TIME	F(1,16) = 574,7	p<5,8e-14
TMS-INTENSITY	F(3,48) = 3,3	p<0.03
FREQUENCY-BAND	F(4,64) = 21626	p<0,0000
TIME*TMS-INTENSITY	F(3,48) = 21,3	p<6,7e-9
TIME*TMS-FREQUENCY	F(5,80) = 6,1	p<0,000077
TIME*FREQUENCY-BAND	F(4,64) = 3152,5	p<0,0000
TMS-INTENSITY*FREQUENCY-BAND	F(12,192) = 70,3	p<0,0000
TMS-FREQUENCY*FREQUENCY-BAND	F(20,320) = 29,7	p<0,0000
TIME*TMS-INTENSITY*TMS-FREQUENCY	F(15,240) = 1,9	p<0,03
TIME*TMS-INTENSITY*FREQUENCY-BAND	F(12,192) = 93,8	p<0,0000
TIME*TMS-FREQUENCY*FREQUENCY-BAND	F(20,320) = 40,1	p<0,0000
TMS-INTENSITY*TMS-FREQUENCY*FREQUENCY-BAND	F(60,960) = 7,0	p<0,0000
TIME*TMS-INTENSITY*TMS-FREQUENCY*FREQUENCY-BAND	F(60,960) = 7,7	p<0,0000

### Stimulation frequency and stimulation intensity thresholds

In general, the stimulation frequency has no significant effect on the band power. But for a stimulation frequency of 0.5 Hz, we stated a selective influence on the delta, theta, alpha and gamma band.

**Figure 2:**
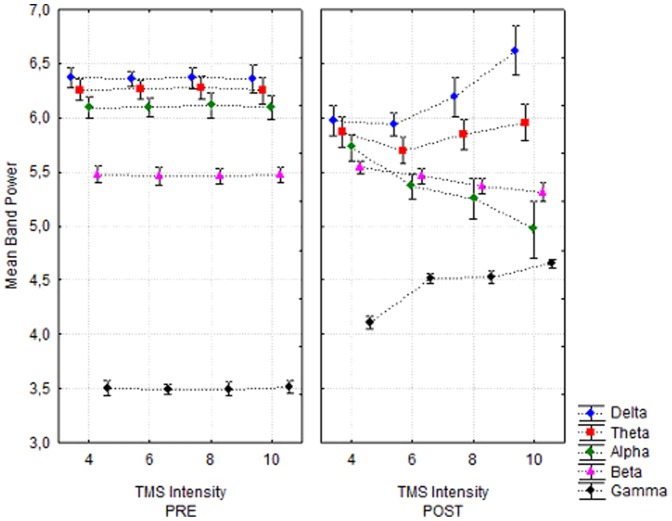
Interaction of TIME*TMS-INTENSITY*FREQUENCY_BAND. Mean band power values are given in V/√Hz and represent an average over the runs.

The stimulation intensity has strong, but varying effects on the band power in the frequency bands. In the delta band, we observed a decreased band power for the stimulation intensities 4, 6, and 8 but an increase for a stimulus intensity of 10 compared to the mean band power before TMS (Fig. 2). The theta band power and the alpha band power were lower compared to the band power before TMS stimulation. The mean band power of the alpha band decreased linearly with increasing stimulation intensity (Fig. 2). The gamma band power was always larger after TMS stimulation, an effect that became more pronounced with increasing TMS intensity.

### Stimulation intensity as dominant parameter

**Figure 3:**
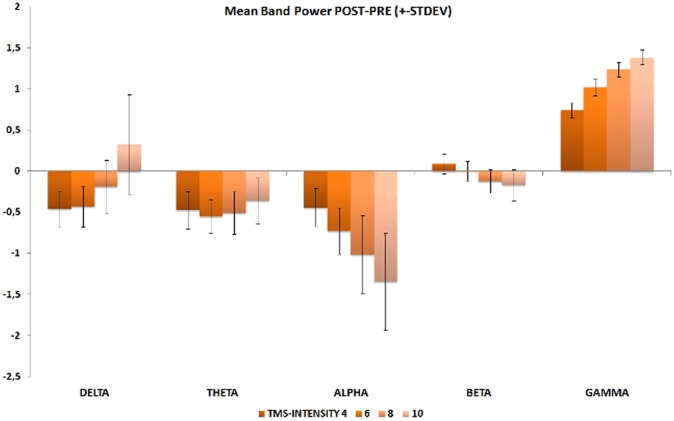
Difference of mean band power for factor TIME: POST – PRE. Data were averaged separately over all segments and separately for TMS-intensity over all TMS frequencies except 0.5 Hz for POST. Error bars indicate standard deviation of the factor POST.

Ignoring the stimulation frequency of 0.5 Hz, we observed a linear decrease of the alpha band power (Pearson product moment correlation: r = −1 p<0.05) and beta band power (r = −0.99, p<0.05) and a linear growth of the gamma band power (r = 0.99, p<0.05) while the power of the theta band (r = 0.6) did not show a statistically significant linear behaviour (Fig. 3).Thus, as a main result, our simulation revealed that the effects of rTMS on the alpha, delta and gamma band depend linearly on the applied stimulus intensity.

Concerning the stimulation intensity, we observed significant changes of the band power (Fig. 2). The change in intensity either caused an increased or decreased band power in the alpha, gamma, theta and delta band, whereas in the beta band, the band power is not changed (Fig. 2).

## Discussion

Our model turns out to be beneficial for explaining the experimentally observed effects of rTMS mentioned in the related work section for the following reasons. First, it integrates the effect of rTMS into the neural model by considering rTMS as an external input source. Second, we work with the summed output of all neurons (eLFP) which we consider to be comparable to the local field potential (LFP) for two reasons: a) we defined a network of interconnected excitatory and inhibitory neurons, whose synaptic activity lead to local changes of the electric field, and b) we neither included long range connections from or to different networks nor did we look at the action potentials of a small subset of neurons which would be compatible to physiological measures like multi-unit activity (MUA). Third, we fit the model with experimentally validated parameter values [22], [23].

Regarding the choice of our model, we consider a random graph [24] network based on McCulloch-Pitts neurons as favorable for the examination of rTMS effects on the band power, because we aim at modelling the influence of rTMS on the brain area directly affected by the magnetic field of the TMS coil. The random graph model for the generation of the graph is the G(n,p) model proposed by [24], which is not differing from the exponential graph models (ERGM) if the edges metric constitutes the only network metric [17] . We also prefer the simple McCulloch-Pitts neurons, since a complex model for the behaviour of the neurons based on differential equations like the Hodgkin-Huxley model [25] often exceeds, beyond numerical problems, the limits of practical computability.

The network size has no statistical relevance. This scale-freeness of the model has important consequences: a) the results can be transferred on networks of different sizes and b) we can predict local field potentials, cortical potentials and even potentials on the surface of the head. This complies with experimental findings that indicate the independence of scale [26], i.e. microelectrode recordings of the activity of a small number of neurons and scalp electrode recordings reflecting the activity of millions of neurons are essentially identical.

The results of the simulation itself aim at identifying how band power depends on rTMS frequency and intensity, and, in doing so, advance the development of rTMS as an efficient therapeutic treatment for mental disorders [27] and diseases like tinnitus [28].

The results replicate *in vivo* experimental observations in three main ways: gamma band power is increased, alpha band power depends on rTMS intensity, and band power is independent of rTMS frequency in most protocols. The model also reveals the stability of the band power in the beta band.

We observe an increase in the gamma band power for all stimulus intensities and frequencies above 0.5 Hz. These findings agree with *in vivo* experimental results [13], [14] and imply that short rTMS pulses at repetition frequencies greater than 0.5 Hz significantly enhance gamma band activity. No experimental results have been performed with a 0.5 Hz stimulus protocol, and thus this cut-off frequency can neither be confirmed nor substantiated by experiments.

In the alpha band, the simulation shows that changes strongly depend on rTMS intensity. This observation accounts for contradictory experimental results, regarding the effect of rTMS on the power in the EEG alpha band [8], [10], [11].

As seen in recent research studies and confirmed by these findings, the rTMS intensity does significantly affect the EEG power across all frequency bands. The stimulation intensity influences the network activity by causing a higher number of spikes at the neuronal level and accordingly changes of the eLFP with increasing intensity. It is important to note that the effects are studied on a neuronal network level which means that our model neglects potential side effects of the applied rTMS energy.

This model does not explain all experimentally observed results of rTMS. Our simulation and most *in vivo* experiments do not reveal information about long-term effects of rTMS on the neuronal level e.g. changes of synapses like synaptic plasticity or synchronization of brain activity. This point must be investigated further to predict the efficiency of rTMS therapies.

In addition, we assume a uniform perturbation of the network by the rTMS stimulation. It is left open how the stimulation affects remote brain areas not directly affected by the magnetic field. The effects of rTMS on remote, not directly stimulated cortical areas are not included in this model. We believe that first and foremost, it is vital to understand the effects of rTMS on the directly stimulated brain areas and to close the gap between model behaviour and EEG measurement data.

Our model for rTMS experiments offers an opportunity to corroborate and predict experimental results, which can be used to further develop the model. This approach could be extended to allow the development of models that depict pathological activity like depression, schizophrenia or epilepsy and the effects of rTMS on abnormal brain activity, representing an important preliminary step towards the clinical use of rTMS.

## Methods

Our simulation is based on a random network [24] of neurons implemented as McCulloch-Pitts neurons [29].

The discrete time evolution for each neuron is given by the input function I_i_(t) and the output function E_i_(t) with a threshold value of Θ_i_ = −60 mV for the generation of an action potential (AP), and with constant c denoting the change in postsynaptic potential triggered by the arrival of an AP at the synaptic cleft [22]. Firing neurons either cause a hyperpolarisation or depolarization of ± (0.2 mV–1 mV) [22]. For sake of simplicity, we assume a value of ±0.5 mV for the effect of inhibitory and excitatory postsynaptic potentials on the cell membrane and set c = 0.5. The effects of a single AP on the postsynaptic membrane potential is not strong enough to generate an AP since the membrane potential returns to its resting potential of −70 mV after a few milliseconds [22]. The net effect of spatial summation depends on various parameters [22]. In the model, we assume a linear spatial and temporal summation.

Whether the synapses are of the inhibitory or the excitatory type is coded in the randomly generated adjacency matrix a_ij_ [24]. The number of synapses depends on the connection probability in the network which in turn determines the average node degree k of each neuron in the network [24]. Existing experimental results could be explained best for k = 120. Table 2 shows the connection probability p resulting in k = 120 for different network sizes. These theoretical considerations are substantiated by microscopic analysis of mammal cortical tissue indicating a connection probability of p = 0.12 for a cluster of 1,000 neurons and a ratio of 80∶20 between excitatory and inhibitory synapses [23].

**Table 2:** Connection probability and network size.

Network size	Connection probability p	Average number of neighbours k
1000	0.8799	120
2000	0.9400	120
4000	0.9700	120
8000	0.9850	120
16000	0.9925	120

**Figure 4:**
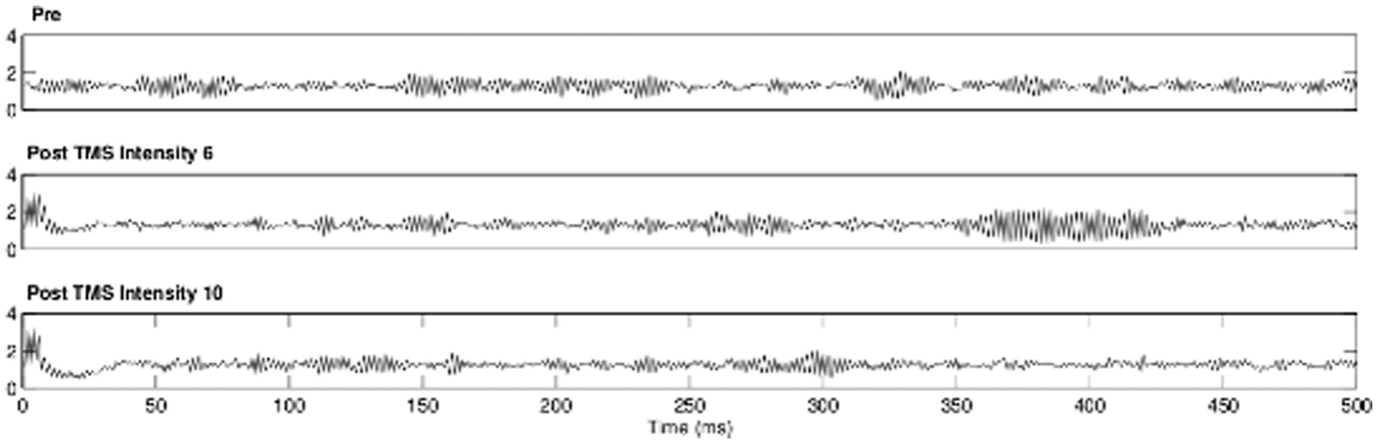
Typical time course of the output function ρ(t) of the neural network 500 ms before the stimulation (PRE) and 500 ms after the last TMS pulse (POST) for the two intensities TMS-INTENSITY 6 and TMS-INTENSITY 10.

For determining the stimulus intensities, we assume that a single TMS pulse triggers the simultaneous depolarization of neurons [30]. Numerical analysis of our network revealed a mean change of 1.25 mV of the synaptic potential per neuron for k = 120. Thus, we set the TMS induced disturbance to 160%, 240%, 320% and 400% of the mean change of the synaptic potential which corresponds to a change of 2 mV, 3 mV, 4 mV, 5 mV. One can think of that as the increase caused by the simultaneous spiking of either 4 neighbours (TMS-INTENSITY 4), 6 neighbours (TMS-INTENSITY 6), 8 neighbours (TMS-INTENSITY 8) or 10 neighbours (TMS-INTENSITY 10) [30]. These values guarantee stable oscillatory behaviour of the network after the TMS stimulation for all network sizes. The TMS stimulation itself is applied within a period of 7.99 s, beginning at 8,000 s and lasting to 15,999 s. The simulation output is defined as the sum of the output E_i_(t) of all neurons at time t which corresponds to macroscopic properties like the LFP or the surface EEG and will be called equivalent local field potential (eLFP). For the processing of the output ρ(t) (Fig. 4) of the model, we selected one segment of 1 s ending immediately before the first (PRE) and one segment of 1 s beginning after the last stimulation (POST). For all combinations of the model parameters TMS-INTENSITY and TMS-FREQUENCY we calculated 20 independent runs, each of the runs incorporating a new, random adjacency matrix (this lead to 4 * 6 = 24 models times 20 independent runs which leads to 480 model runs). In rare cases ρ(t) showed limit cycle behaviour for certain model runs, which were treated as missing values. In this case the independent run was excluded from the statistics. This left us with 17 independent runs as a source of variance of ρ(t). As the variance originates from the adjacency matrix, which reflects individual interconnection of the neurons, we argue that this would also reflect variations of the network likely to be found in different cortical columns within the same individual or even across individuals.

The spectral power of the output ρ(t) is calculated using the Fast-Fourier-Transform (FFT) and averaged within the common frequency bands (delta: 0.5–4 Hz, theta: 4–8 Hz, alpha: 8–12 Hz, beta: 12–24 Hz, gamma: 24–48 Hz). The estimates of the band power are statistically analysed using STATISTICA 6.1. We perform a repeated measures ANOVA with band power as dependent variable for the 17 runs (random factor), the repeated measures factor TIME (2 steps: PRE, POST), and 3 fixed factors: TMS-FREQUENCY (6 steps: 0.5 Hz, 1 Hz, 2 Hz, 5 Hz, 10 Hz, 20 Hz), TMS-INTENSITY (4 steps: 4, 6, 8, 10) and FREQUENCY-BAND (5 steps: delta, theta, alpha, beta, gamma).
